# Factors Contributing to Negative Outcomes Associated with Medications and Drug-Related Problems in Kidney Replacement Therapy—A Hospital-Based Prospective Observational Study

**DOI:** 10.3390/jcm13041048

**Published:** 2024-02-12

**Authors:** Alfonso Pereira-Céspedes, Alberto Jiménez-Morales, Aurora Polo-Moyano, Magdalena Palomares-Bayo, Fernando Martínez-Martínez, Miguel Ángel Calleja-Hernández

**Affiliations:** 1 Pharmaceutical Care Research Group, Pharmacy Faculty, University of Granada, 18071 Granada, Spain; alberto.jimenez.morales.sspa@juntadeandalucia.es (A.J.-M.); femartin@ugr.es (F.M.-M.); mangel.calleja.sspa@juntadeandalucia.es (M.Á.C.-H.); 2 Pharmacy Department, Hospital Universitario Virgen de las Nieves, 18014 Granada, Spain; 3 Centro Nacional de Información de Medicamentos, Instituto de Investigaciones Farmacéuticas, Pharmacy Faculty, University of Costa Rica, San José 11501-2060, Costa Rica; 4 Nephrology Department, Hospital Universitario Virgen de las Nieves, 18014 Granada, Spain; auroreja2002@yahoo.es (A.P.-M.); mapalomabayo@gmail.com (M.P.-B.); 5 Pharmacy Department, Hospital Universitario Virgen Macarena, 41009 Seville, Spain

**Keywords:** kidney replacement therapy, medication review, drug-related problems, negative outcomes associated with medication

## Abstract

Background: Negative outcomes associated with medications (NOM) and drug-related problems (DRP) significantly impact individuals with kidney replacement therapy (KRT) given the complexities of managing kidney disease and associated comorbidities. The present study aims to assess the frequency of NOMs/DRPs among KRT patients and identify contributing factors. Methods: A cross-sectional study was conducted at Virgen de las Nieves University Hospital (Granada, Spain), involving 117 outpatient adults with KRT. Data were collected from February 2021 to July 2023 using electronic records, semi-structured interviews (Dáder Method), and discussions with nephrology specialists. NOMs/DRPs were identified following treatment guidelines. Binary logistic regression was used to determine associated factors (*p*-value < 0.05). Results: Across 117 patients, 2436 NOMs and 3303 DRPs were identified, averaging 20.82 NOMs and 28.23 DRPs per patient. Prevalent NOMs included untreated conditions (58.95%), quantitative ineffectiveness (35.43%), and non-quantitative safety problems (5.13%). Dominant DRPs were undertreated conditions (37.63%), wrong dose/posology/length (33.00%), risk of adverse drug reactions (ADR) (16.14%), and non-adherence (6.87%). Patients with ADR, undertreated conditions, and anemia were associated with quantitative ineffectiveness. Risk of ADR and vitamin D deficiency/insufficiency correlated with non-quantitative safety problems. Conclusions: KRT patients exhibited a substantial prevalence of NOMs/DRPs. Further research is needed to deepen our understanding of these complexities for improved patient care.

## 1. Introduction

Chronic kidney disease (CKD) stands as a formidable global public health challenge, demanding timely identification and intervention to impede its progression [1,2,3,4,5]. Upon reaching the critical stage culminating in end-stage renal disease (ESRD), patients confront the necessity for kidney replacement therapies (KRT), encompassing hemodialysis (HD), peritoneal dialysis (PD), and kidney transplant (KT) [1,5]. The anticipated number of individuals grappling with ESRD requiring KRT is projected to span from 4.902 to 7.083 million worldwide [1].

The intricacies inherent in KRT present formidable medical challenges, compelling the use of a plethora of medications to address associated comorbidities [6]. These health conditions, in turn, impose substantial financial strains on healthcare systems. Individuals grappling with these ailments are susceptible to issues related to medications, encapsulated as drug-related problems (DRP), which, in turn, heighten the risks of morbidity, mortality, and healthcare expenditures [1,4,5,7,8]. The resultant negative outcomes associated with medications (NOM) further compound these challenges [1,7,8].

Despite the critical nature of the subject, limited studies focusing on NOMs and associated factors in the context of KRT have been published [7]. Recognizing NOMs and DRPs emerges as a pivotal responsibility that pharmacists can effectively manage in collaboration with other healthcare professionals through comprehensive medication reviews and subsequent follow-ups [1,3,7,8,9]. The evaluation of NOMs/DRP within the KRT population in Spain holds significance for a multitude of reasons, including the escalating prevalence of CKD within the local community. This increase in prevalence has the potential to detrimentally impact quality of life, prolong hospitalization durations, and elevate the likelihood of both mortality and morbidity [10]. Consequently, this study aims to elucidate the frequency of NOMs/DRPs and associated factors among patients undergoing KRT.

## 2. Materials and Methods

### 2.1. Design and Setting

A cross-sectional and prospective observational study was carried out at the nephrology department of Virgen de las Nieves University Hospital (Granada, Spain). The research spanned from 2 February 2021 to 31 July 2023, encompassing a period of 29 months, and focused on patients with ESRD.

### 2.2. Study Population and Eligibility Criteria

All outpatient individuals meeting the following criteria were considered for inclusion in the study: being over 18 years old; undergoing KRT such as HD, PD, or KT at nephrology department during the study period; and expressing a willingness to participate. Patients with cognitive impairment and inpatients were excluded from the study due to their medical conditions. 

No cluster randomization was performed during the study. All patients meeting the inclusion criteria for the study were recruited by nephrology experts in clinical practice.

The stage of kidney disease was determined by categorizing patients using the estimated Glomerular Filtration Rate, calculated from serum creatinine levels employing the Chronic Kidney Disease Epidemiology Collaboration (CKD-EPI) equation, as documented in the electronic medical records. Laboratory tests were recorded in the electronic medical record as a standard component of routine clinical practice.

### 2.3. Methods

Data collection involved two main sources: electronic medical records and semi-structured interviews following the Dáder Method developed by the Pharmaceutical Care Research Group at the University of Granada [11]. Additionally, discussions with nephrology experts were facilitated by pharmacists.

The pharmacist conducted a thorough review of electronic medical records and conducted semi-structured interviews. These interviews aimed to assess the number of prescribed medications and identify baseline demographics, comorbidities, clinical laboratory data, allergies, and the number of NOMs/DRPs.

All the data for the analysis, extracted from the medical records, treatment chart, laboratory data, and by interviewing patients, were recorded onto a data collection form.

### 2.4. Study Outcomes

The primary outcome was the prevalence of NOMs/DRPs and associated factors among outpatients undergoing KRT. These included different types of NOMs/DRPs, as was reported in previous studies according to the Granada Third Consensus [11], and associated factors such as age, sex, number of comorbidities, number of medications, clinical laboratory data, time in KRT, time in KT and number of hospitalizations. 

Additional secondary outcomes include types of medications and comorbidities.

### 2.5. Sample Size

We included all patients consulting the nephrology department of the Virgen de las Nieves University Hospital and meeting the eligibility criteria.

The sample size was calculated by using simple proportion formula with the estimated prevalence of DRP among CKD patients, *p* = 81.5% [8], 95% confidence interval, and sample error of 5%, *n* = 231. In total, 170 ESRD patients were admitted to the hospital. The final sample size, 117, was calculated using the correction formula.

### 2.6. Operational Definitions

#### 2.6.1. NOMs

A NOM is a result affecting the health of the patient that is or may be associated with the use of medications [11].

#### 2.6.2. DRPs

A DRP is an event or circumstance involving drug therapy that actually or potentially interferes with desired health outcomes [11].

### 2.7. Statistical Analysis

The prevalence of NOMs/DRPs is presented as a percentage, with a 95% confidence interval using an exact binomial test. We tabulated descriptive statistics by a count or percentage for categorical variables, and the mean ± standard deviation (SD), median, or interquartile range (IQR), when appropriate, was used for continuous data.

Spearman’s Rank correlation (r_s_) was used to measure the association or correlation between the number of NOMs as the dependent variable and the following independent variables: number of DRPs, number of comorbidities, number of medications, age, clinical laboratory data, time in KRT, time in KT and number of hospitalizations. A chi-square test of independence was performed to examine the relationship between categorical variables. A *t*-test was performed to compare means (baseline and final) for continuous data.

Binary logistic regression was used to analyze the association between independent and dependent variables, and variables with a *p*-value <0.05 were a candidate for multivariate analysis. 

Statistical tests were conducted using a 5% level of significance. All analyses were conducted using SPSS 29.0.1.1(171) (IBM Corporation, Chicago, IL, USA, 2023) and R software version 4.3.2 (31 October 2023).

### 2.8. Ethics

Institutional review board approval was obtained from the Andalusian Biomedical Research Ethics Committee (FIS-IRB-2020-01) on 28 July 2020. Written informed consent was obtained from each study participant.

### 2.9. Data Management

The data were arranged into folders identified by patient ID, encompassing patient demographics and additional variables. Variable codes were documented in a separate file, and metadata were supplied to clarify the data format and abbreviations. The information was securely stored on a password-protected server, accessible exclusively to investigators. Routine backups were performed, and access was restricted to the research team to uphold confidentiality.

In compliance with Spanish legislation, the data will be retained for five years post the study’s completion. Personal identifiers were eliminated, and a distinct study ID was employed to de-identify patient records, guaranteeing anonymity.

## 3. Results

### 3.1. Sociodemographic and Clinical Characteristics of the Study Population

From patient data obtained during a 29-month study period, 117 KRT patients were included in the study. Eleven patients died throughout the study due to complications of kidney disease (Table 1).

In terms of the clinical characteristics of the patients, the cause of end-stage renal disease was unknown in 26.50% of cases, while glomerulonephritis accounted for 23.93% (refer to Table 2).

Primary comorbidities of note include mineral and bone disorder (91.45%), anemia (89.74%), and arterial hypertension (86.32%) (see Table 3). 

Additional clinical parameters are presented in Table 4. A *t*-test was conducted to examine the means (baseline and final) of the clinical parameters of interest throughout the follow-up period. The results indicated a statistically significant difference in the means (baseline and final) of urea (*p* < 0.01) and HDL cholesterol (*p* < 0.05).

### 3.2. Types of NOMs/DPRs

During the study duration, 117 patients with KRT exhibited a total of 2436 NOMs (Table 5) and 3303 DRPs (Table 6). This equates to an average rate of 20.82 NOMs and 28.23 DRPs per patient.

A *t*-test was conducted to assess the means (baseline and final) of NOM occurrences in the patient group. The analysis revealed no statistically significant differences between the means (baseline and final) of NOM, including undertreated health problems, effect of unnecessary medicine, non-quantitative ineffectiveness, quantitative ineffectiveness, non-quantitative safety, and quantitative safety.

A *t*-test was conducted to assess the means (baseline and final) of DRP in the patient group, as indicated in Table 6. A statistically significant difference was observed between the means (baseline and final) of DRPs categorized under risk of adverse effects (*p* < 0.01). However, no statistically significant difference was noted between the means (baseline and final) of the following DRPs: personal characteristics, wrong dose/posology/length, lack of knowledge of medication use, unnecessary drug, non-adherence to medication, other conditions affecting the treatment, precautions, undertreated condition, and others.

### 3.3. Medications

At the baseline, patients used a total of 1398 medications, out of which 1222 were administered at home. By the end of the study period, the overall medication count increased to 1460, with 1279 medications administered at home (Table 7). No statistically significant differences were found between the medians of total medications administered at home and during the dialysis process at baseline and at the end of the study.

The predominant instance of quantitative ineffectiveness, characterized by insufficient prescribed quantity or dosage of medication, was observed with darbepoetin alfa, accounting for 94 cases (97.9%). To examine the potential association between quantitative ineffectiveness in NOM and darbepoetin alfa, a chi-square test of independence was performed (X^2^(1, *n* = 117) = 6.27, *p* < 0.05) (OR = 7.62, 95% CI = 0.814–97.38).

In total, 64.4% (47 cases) demonstrated non-compliance with sevelamer, while 66.7% (22 cases) exhibited non-adherence to calcium polystyrene sulfonate. A chi-square test of independence was performed to investigate the statistically significant relationship between non-adherence and sevelamer (X^2^(1, *n* = 117) = 6.12, *p* < 0.05) (OR = 2.589, 95% CI = 1.131–6.057). Additionally, a chi-square test of independence was executed to explore the association between non-adherence and calcium polystyrene sulfonate (X^2^(1, *n* = 117) = 2.30, *p* = 0.13) (OR = 1.897, 95% CI = 0.767–4.911).

### 3.4. Factors Influencing NOMs/DRPs

The total DRPs, specifically the risk of adverse effects (adverse drug reactions, ADR) (r_s_ = 0.314; *p* < 0.001), wrong dose/posology/length (r_s_ = 0.344; *p* < 0.001), non-adherence to medication (r_s_ = 0.244; *p* = 0.008), undertreated conditions (r_s_ = 0.912; *p* < 0.001), and the total number of comorbidities (r_s_ = 0.395; *p* < 0.001), exhibited significant associations with NOMs, specifically the number of untreated health problems.

The number of the following DRPs: risk of adverse effects (r_s_ = 0.389; *p* < 0.001), individual characteristics (r_s_ = 0.311; *p* < 0.001), wrong dose/posology/length (r_s_ = 0.730; *p* < 0.001), and undertreated conditions (r_s_ = 0.299; *p* < 0.05); demonstrated significant associations with the number of NOMs, specifically quantitative ineffectiveness. Conversely, urea (r_s_ = 0.322; *p* < 0.001), creatinine (r_s_ = 0.231; *p* < 0.05), calcium (r_s_ = −0.196; *p* < 0.05), phosphorus (r_s_ = 0.401; *p* < 0.001), parathyrin (r_s_ = 0.413; *p* < 0.001), ferritin (r_s_ = 0.254; *p* < 0.01), erythrocytes (r_s_ = −0.198; *p* < 0.05), hemoglobin (r_s_ = −0.250; *p* < 0.01), hematocrit (r_s_ = −0.250; *p* < 0.01), and platelets (r_s_ = −0.211; *p* < 0.05) were significantly associated with the number of NOMs, specifically quantitative ineffectiveness.

Moreover, the number of the following DRPs: risk of adverse effects (r_s_ = 0.451; *p* < 0.001), wrong dose/posology/length (r_s_ = 0.215; *p* < 0.05), and undertreated conditions (r_s_ = 0.184; *p* < 0.05); was significantly associated with the number of NOMs, specifically non-quantitative safety. On the contrary, albumin (r_s_ = −0.186; *p* < 0.05) and magnesium (r_s_ = −0.194; *p* < 0.05) were significantly associated with the number of non-quantitative safety.

A chi-square test of independence was conducted to explore the significant relationship between the undertreated condition in NOM and the following variables: the presence of wrong dose/posology/length (X^2^(1, *n* = 117) = 28.49, *p* < 0.05) and the presence of undertreated conditions (X^2^(1, *n* = 117) = 38.33, *p* < 0.05).

Similarly, a chi-square test of independence was used to investigate the significant association between non-quantitative ineffectiveness in NOM and hyperphosphatemia (X^2^(1, *n* = 117) = 7.68, *p* < 0.05).

Furthermore, chi-square tests of independence were conducted to assess the significant relationships between quantitative ineffectiveness in NOM and the following variables: the presence of wrong dose/posology/length (X^2^(1, *n* = 117) = 92.77, *p* < 0.01), the presence of undertreated conditions (X^2^(1, *n* = 117) = 29.29, *p* < 0.05), and anemia (X^2^(1, *n* = 117) = 14.04, *p* < 0.05).

Additionally, a chi-square test of independence was performed to investigate the significant relationship between non-quantitative safety in NOM and the following variables: the presence of wrong dose/posology/length (X^2^(1, *n* = 117) = 4.83, *p* < 0.05), the presence of risk of adverse effects (X^2^(1, *n* = 117) = 11.15, *p* < 0.05), and vitamin D deficiency/insufficiency (X^2^(1, *n* = 117) = 4.68, *p* < 0.05).

We examined the relationship between independent variables and dependent variables (non-quantitative ineffectiveness, quantitative ineffectiveness, and non-quantitative safety) through the application of both univariate and multivariate logistic regression methods.

In the univariate logistic regression analysis, hyperphosphatemia was found to be associated with non-quantitative ineffectiveness. Additionally, the risk of adverse effects, undertreated conditions, and anemia showed associations with quantitative ineffectiveness. Moreover, the risk of adverse effects and vitamin D deficiency/insufficiency were linked to non-quantitative safety.

Variables with a *p*-value < 0.05 in bivariate analysis were subsequently included in the multiple logistic regression. The results of the multivariate analysis revealed that participants with hyperphosphatemia were 0.08 times more likely to experience non-quantitative ineffectiveness compared to those without hyperphosphatemia (OR = 0.0845, 95% CI: 0.0044–0.5192, *p* = 0.02). 

Moreover, participants with a risk of adverse effects were 1.802 times more likely to have NOM compared to those without a risk of adverse effects (OR = 1.802, 95% CI: 0.076–18.631). Participants with undertreated conditions were 23.883 times more likely to have NOM compared to those without undertreated conditions (OR = 23.883, 95% CI: 1.265–1062.966). Participants with anemia were 8.836 times more likely to experience NOM compared to those without anemia (OR = 8.836, 95% CI: 0.851–90.667) (Table 8).

Participants with a risk of side effects were 14.625 times more prone to have NOM of non-quantitative safety compared to those without the risk of side effects (OR = 14.625, 95% CI: 2.646–273.914, *p* = 0.012). Moreover, participants with vitamin D deficiency/insufficiency were 2.177 times more likely to encounter NOM of non-quantitative safety compared to those without vitamin D deficiency/insufficiency (AOR = 2.177, 95% CI: 0.954–5.058, *p* = 0.06).

### 3.5. Types of Interventions for Preventing or Resolving NOMs/DRPs among KRT Patients

Throughout the 29-month study period, healthcare providers conducted a total of 3355 interventions aimed at addressing issues associated with medication usage. On average, there were 1.4 interventions per NOM and 1.0 intervention per DRP. The most prevalent interventions included dose modification (997 interventions; 29.72%), addition of a new medication (830 interventions; 24.74%), patient health education (580 interventions; 17.29%), medication withdrawal (318 interventions; 9.48%) and schedule modification (26 interventions; 0.77%). Among patient health education interventions, the most frequent were related to non-pharmacological advice (376 interventions; 11.21%), medication adherence (130 interventions; 3.87%) and the use and administration of medicines (74 interventions; 2.20%).

Pharmacists provided health education interventions to the 117 patients, constituting 3.49% of the interventions. On average, there were 1.0 pharmacist interventions per patient. These interventions were accepted in 85.5% of cases, totaling 100 accepted interventions.

## 4. Discussion

Individuals undergoing KRT face an escalated risk of encountering DRPs and NOMs. This heightened susceptibility can be attributed to the presence of comorbidities, complications, and the intricate nature of their medication regimens [1,4].

Our study, which examined 117 patients within the nephrology department, revealed a mean prescription of 12.14 medications and 18.64 comorbidities per patient. Notably, these findings align with those reported in studies conducted in Ethiopia [4], India [12], and the USA [1], where individuals are prescribed an average of 10–12 medications per day, contributing to complex dosing routines and an increased likelihood of encountering DRPs.

The investigation unveiled that antithrombotic agents (94.02%) and COVID-19 vaccines (98.07%) were the most frequently prescribed classes of drugs, while mineral and bone disorder (91.45%) and anemia (89.74%) emerged as the predominant comorbidities. 

A total of 2436 NOMs and 3303 DRPs were identified, with the most common NOMs being untreated health problems (58.95%) and the prevalent DRPs being undertreated conditions (37.63%) and wrong dose/posology/length (33.00%). The rates of NOMs and DRPs were 20.82% and 28.23%, respectively, showcasing the substantial burden within this patient population. Notably, our study adds to the existing literature, as some studies primarily focused on DRPs [4,12] while others exclusively identified NOMs [1,7].

Our analysis of NOMs revealed a significant need for additional drug therapy (58.94%), deviating notably from rates reported in other regions. The most common NOM identified was an untreated health problem (99.15%), contrasting with the prevailing DRP, which was a poorly treated health problem (97.44%). Furthermore, ineffective drug therapy contributed to 35.92% of all NOMs identified, indicating a noteworthy divergence from studies conducted in Ethiopia, India, Canada, Beirut, and the USA [8].

Nonadherence to medicines (55.55%) and adverse drug reactions (ADR) (53.85%) were predominant DRPs in our study, differing significantly from rates reported in Nepal (25% nonadherence), Palestine (19% nonadherence), Greece (60 patients reporting ADR), Germany (8.5% severe drug events), and the United Arab Emirates (14 ADR in 130 CKD patients). Our findings highlight the need for tailored interventions by healthcare professionals, including pharmacists and nephrologists, involving pharmacological and non-pharmacological approaches to mitigate NOMs/DRPs [1,10,13,14,15,16,17,18].

Moreover, our study identified statistically significant correlations between various DRPs and NOMs, emphasizing the complex interplay between these factors. Notably, parameters such as urea, creatinine, potassium, calcium, phosphorus, parathyroid hormone, ferritin, erythrocytes, hemoglobin, and hematocrit exhibited significant associations with NOMs related to quantitative ineffectiveness. These findings enrich the understanding of the intricate relationships between patient parameters and NOMs, shedding light on potential avenues for targeted interventions.

While our study contributes valuable insights, it is essential to acknowledge its limitations. The identification of NOMs lacked the establishment of causal relationships, relying on information retrieved from medical records and patient interviews. Future research endeavors should strive to overcome such limitations to enhance the robustness of conclusions drawn from similar investigations.

In summary, our study underscores the multifaceted nature of NOMs and DRPs among KRT patients, emphasizing the need for tailored interventions, collaborative healthcare approaches, and continued research efforts to advance our understanding and enhance patient outcomes within this complex medical landscape.

## 5. Conclusions

In conclusion, our study reveals compelling findings regarding the prevalence of NOM and DRP among patients undergoing KRT within the nephrology department. The average rates of NOM and DRP per KRT patient were determined to be 20.82 and 28.23, respectively. Notably, factors such as ADR, undertreated conditions, anemia, and vitamin D deficiency were identified as associated contributors to these outcomes.

The intricate nature of the medication regimen, coupled with the unique disease status of these patients, emerged as a pivotal determinant contributing to the heightened incidence of NOMs. It is evident that this specific patient population demands meticulous attention, emphasizing the importance of a comprehensive healthcare approach. A thorough medication review, sustained follow-up, and collaborative care from all pertinent healthcare professionals are imperative to mitigate complications stemming from the administered therapies and healthcare interventions. These insights underscore the necessity for a multidisciplinary approach to optimize patient outcomes and enhance the overall quality of care for individuals undergoing KRT.

## Figures and Tables

**Table 1:** Characteristics of KRT patients at the Virgen de las Nieves University Hospital (Granada, Spain).

Baseline Characteristics	*n* = 117 ^a^
Age, mean (SD)	63 (14) years
Age > 75 years	31 (26.50)
Age > 60 years	71 (60.68)
Sex (Males)	62 (52.99)
Current renal replacement therapy	
Hemodialysis	72 (61.54)
Deceased donor	28 (23.93)
Peritoneal dialysis	16 (13.68)
Living donor	1 (0.85)
Personal history of renal transplant	23 (19)
Personal history of hemodialysis	35 (29.91)
Personal history of peritoneal dialysis	28 (23.93)
Number of renal transplants, mean (SD)	0.55 (0.71)
Time in dialysis, median (IQ)	41 (85.50–19) months
Time in kidney replacement therapy, median (IQ)	50 (105–31) months
Time in kidney transplant, mean (SD)	26 (91) months
Number of hospitalizations, mean (SD)	0.20 (0.44)
Number of comorbid conditions per patient, mean (SD).	18.64 (4.35)
Number of medications per patient, median (IQ)	12 (13–10)
Number of medications per patient, median (IQ) (end of study)	12 (15–10)
Number of medications administered at dialysis per patient, median (IQ)	2 (3–0)
Number of medications administered at dialysis per patient, median (IQ) (end of study)	1 (3–0)
Number of medications administered at home per patient, median (IQ)	10 (12–8)
Number of medications administered at home per patient, median (IQ) (end of study)	10 (14–9)
Allergies to medications	34 (29.06)

^a^ Unless otherwise indicated: numbers present *n* (%). Kidney replacement therapy (KRT).

**Table 2:** Underlying cause of End-Stage Renal Disease of KRT patients at the Virgen de las Nieves University Hospital (Granada, Spain).

Underlying Cause of End-Stage Renal Disease	*n* = 117 ^a^
Unknown	31 (26.50)
Glomerulonephritis	28 (23.93)
Diabetic nephropathy	16 (13.68)
Polycystic kidney disease	12 (10.26)
IgA nephropathy	9 (7.69)
Systemic disease	5 (4.27)
Vascular nephropathy	5 (4.27)
Reflux nephropathy	4 (3.42)
Pyelonephritis	3 (2.56)
Nephrotoxicant-induced nephropathy	1 (0.85)
Interstitial nephropathy	1 (0.85)
Congenital anomalies of the kidney and urinary tract	1 (0.85)
Tuberous sclerosis	1 (0.85)

^a^ Unless otherwise indicated: numbers present *n* (%). Kidney replacement therapy (KRT).

**Table 3:** Comorbidities and risk factors for CKD of KRT patients at the Virgen de las Nieves University Hospital (Granada, Spain).

Comorbidities/Risk Factors for CKD ^b^	*n* = 117 ^a^
Diabetes	32 (27.35)
Hypertension	101 (86.32)
Autoimmune diseases	7 (5.98)
Other Systemic infections (e.g., HIV, hepatitis B virus, hepatitis C virus)	53 (45.30)
Cytomegalovirus	6 (5.13)
Exit-site infection	6 (5.13)
Recurrent urinary tract infection	24 (20.51)
Nephrotoxic medications (e.g., nonsteroidal anti-inflammatory drugs, herbal remedies, lithium)	5 (4.27)
Kidney stones	9 (7.69)
Malignant neoplasms	25 (21.37)
Obesity and other hyperalimentation	26 (22.22)
Smoking or personal history of smoking	44 (37.61)
Other cardiovascular diseases	71 (60.68)
Dyslipidemia	83 (70.94)
Personal history of COVID-19	30 (25.64)
Family history of kidney disease	26 (22.22)
Anemia	105 (89.74)
Mineral and bone disorder	107 (91.45)
Hyperphosphatemia	98 (83.76)
vitamin D deficiency/insufficiency	79 (67.52)
secondary hyperparathyroidism	98 (83.76)
Hypocalcemia	64 (54.70)
Hypomagnesemia	47 (40.17)
Hyponatremia	45 (38.46)
Hypoalbuminemia	65 (55.56)
Hyperkalemia	83 (70.94)
Metabolic acidosis	18 (15.38)
Hyperuricemia	52 (44.44)
Diseases of the nervous system	35 (29.91)
Diseases of the respiratory system	64 (54.70)
Diseases of the digestive system, number (%)	95 (81.20)
Diseases of the skin and subcutaneous tissue	32 (27.35)
Diseases of the musculoskeletal system and connective tissue	46 (39.32)
Other diseases of the genitourinary system	8 (6.87)
Other diseases of the blood and blood-forming organs	44(37.61)
Mental and behavioral disorders	41 (35.04)
Disorders of thyroid gland	31 (26.50)

^a^ Unless otherwise indicated: numbers present *n* (%). ^b^ The list of comorbidities is exhaustive for our cohort and was identified as documented in the medical records. Chronic kidney disease (CKD); kidney replacement therapy (KRT).

**Table 4:** Serum clinical laboratory data of KRT patients at the Virgen de las Nieves University Hospital (Granada, Spain).

Serum Clinical Laboratory Data	*n* = 117 ^a^ Mean (SD)	Baseline Mean (SD)	Final Mean (SD)	*p*-Value
Glucose mg/dL	102.53 (19.02)	107.55 (35.65)	107.34 (36.62)	0.954
Urea mg/dL	117.18 (36.46)	117.75 (53.95)	99.76 (45.05)	<0.01
Creatinine mg/dL	7.34 (3.23)	7.23 (3.73)	6.89 (3.88)	0.347
Uric acid mg/dL	5.88 (0.94)	5.63 (1.50)	5.69 (1.54)	0.752
Total Proteins g/L	6.26 (0.59)	6.33 (0.71)	6.41 (0.71)	0.256
Albumin g/L	3.59 (0.41)	3.65 (0.48)	3.63 (0.55)	0.656
Sodium mEq/L	138.58 (2.87)	138.96 (3.12)	138.52 (3.65)	0.210
Potassium mEq/L	4.81 (0.58)	4.93 (0.82)	4.79 (0.79)	0.125
Calcium mg/dL	8.77 (0.55)	8.74 (0.82)	8.82 (0.88)	0.370
Phosphorus mg/dL	4.64 (1.12)	4.44 (1.61)	4.44 (1.48)	0.984
Magnesium mg/dL	2.08 (0.33)	2.09 (0.37)	2.06 (0.41)	0.471
intact parathyrin pg/mL	334.16 (186.88)	306.23 (241.57)	312.22 (281.10)	0.840
Vitamin D (25OH) ng/mL	25.17 (10.84)	23.49 (14.48)	25.76 (14.70)	0.184
Iron ug/dL	73.80 (20.25)	75.16 (35.52)	71.57 (29.14)	0.338
Ferritin ng/mL	490.37 (387.85)	468.69 (460.18)	535.99 (904.84)	0.443
Transferrin mg/dL	189.63 (37.78)	185.58 (50.50)	190.60 (47.63)	0.233
Transferrin (saturation index) %	32.84 (10.10)	33.16 (17.92)	31.81 (15.47)	0.465
Folic acid ng/mL	10.65 (5.95)	10.16 (7.48)	10.82 (7.47)	0.285
Vitamin B12 pg/mL	489.12 (293.31)	454.52 (227.93)	482.33 (256.80)	0.075
Total cholesterol mg/dL	168.23 (31.14)	157.42 (44.99)	151.09 (43.25)	0.111
HDL cholesterol mg/dL	55.33 (19.42)	57.58 (19.63)	54.62 (19.98)	0.032
LDL cholesterol mg/dL	93.74 (25.80)	97.64 (30.99)	94.09 (33.21)	0.407
Triglycerides mg/dL	130.79 (56.25)	129.51 (71.27)	122.25 (61.92)	0.298
C-reactive protein mg/L	13.22 (20.79)	17.63 (53.91)	14.48 (32.85)	0.508
Erythrocytes (count) × 10^6^/uL	3.71 (0.52)	3.77 (0.66)	3.81 (0.64)	0.486
Hemoglobin g/dL	11.50 (1.23)	11.57 (1.90)	11.65 (1.64)	0.693
Hematocrit %	35.02 (3.52)	35.85 (5.53)	36.13 (5.16)	0.626
Leukocyte (count) × 10^3^/uL	7.32 (4.63)	8.86 (17.04)	7.04 (4.25)	0.253
Platelet (count) × 10^3^/uL	193.49 (67.36)	191.03 (67.27)	188.15 (71.58)	0.578

^a^ Unless otherwise indicated: numbers present *n* (%).

**Table 5:** Types of treatments for uncontrolled health problems identified as NOMs among KRT patients.

Types NOMs	*n* = 2436 ^a^ NOMs			
	Baseline, Mean (SD)	Final, Mean (SD)	*n*	*p*-Value
Untreated health problem (requiring additional drugs)	0.64 (1.03)	0.74 (1.03)	1.436 (58.95)	0.468
Effect of unnecessary medicine	0	0	0	-
Non-quantitative ineffectiveness	0	0.02 (0.13)	12 (0.49)	0.158
Quantitative ineffectiveness (prescribed quantity or dosage of a medication is insufficient)	0.36 (0.66)	0.44 (0.82)	863 (35.43)	0.359
Non-quantitative safety problem (adverse drug reactions)	0.03 (0.16)	0.09 (0.31)	125 (5.13)	0.071
Quantitative safety problem	0	0	0	-

^a^ Unless otherwise indicated: numbers present *n* (%); negative outcomes associated with medications (NOM); standard deviation (SD); kidney replacement therapy (KRT).

**Table 6:** Types of DRPs identified as potential causes of ineffectiveness or unsafety in the treatment of uncontrolled health problems among KRT patients.

Types of DRP	*n* = 3303 ^a^ DRP			
	Baseline Mean (SD)	Final Mean (sd)	*n*	*p*-Value
Wrong administration	0	0	10 (0.30)	-
Risk of adverse effects (adverse drugs reactions)	0.77 (0.30)	0.34 (0.92)	533 (16.14)	0.004
Individual characteristics	0.02 (0.18)	0.03 (0.16)	24 (0.73)	0.707
Improper medication storage	0	0	1 (0.03)	-
Contraindication	0	0	3 (0.09)	-
Wrong dose/posology/length	0.39 (0.68)	0.60 (1.33)	1090 (33.00)	0.139
Duplicity	0	0	2 (0.06)	-
Wrong Dispensing	0	0	0	-
Wrong Prescription	0	0	0	-
Wrong use of medication	0	0	2 (0.06)	-
Lack of knowledge of medicines	0	0.01 (0.09)	1 (0.03)	0.319
Medicines Interactions	0	0	1 (0.03)	-
Not necessary drug	0.05 (0.22)	0.06 (0.27)	130 (3.94)	0.798
Non-adherence	0.09 (0.29)	0.17 (0.46)	227 (6.87)	0.129
Other conditions affecting the treatment	0.01 (0.09)	0	10 (0.30)	0.319
Precautions	0.01 (0.09)	0	2 (0.06)	0.319
Undertreated condition	0.60 (1.00)	0.61 (1.02)	1243 (37.63)	0.895
Other DRPs	0.085 (0.09)	0	24 (0.73)	0.319

^a^ Unless otherwise indicated: numbers present *n* (%); drug-related problems (DRP); standard deviation (SD); kidney replacement therapy (KRT).

**Table 7:** Overview of the types of medications used in the treatment of patients with KRT ^a^.

ATC Code	Medication	*n* = 117 ^b^
H05	Anti-parathyroid agents	
H05BX01	Cinacalcet	40 (34.19)
H05BX02	Paricalcitol	64 (54.70)
H05BX04	Etelcalcetide	18 (15.38)
H05BX05	Calcifediol	97 (82.91)
H03AA01	Levothyroxine sodium	21 (17.95)
A11CC	Vitamin D and analogues	
A11CC03	Alfacalcidol	3 (2.53)
V03AE	Drugs for treatment of hyperkalemia and hyperphosphatemia	
V03AE01	Polystyrene sulfonate	39 (34.21)
V03AE02	Sevelamer	73 (62.39)
V03AE03	Lanthanum carbonate	14 (11.97)
V03AE04	Calcium acetate and magnesium carbonate	18 (15.38)
V03AE05	Sucroferric oxyhydroxide	17 (14.53)
V03AE07	Calcium acetate	24 (20.51)
V03AE09	Patiromer calcium	2 (1.71)
V03AE10	Sodium zirconium cyclosilicate	16 (13.68)
B03XA	Other antianemia preparations	
B03XA02	Darbepoetin alfa	96 (82.05)
B03	Iron preparations	84 (73.68)
A02	Drugs for acid related disorders	102 (87.18)
A10A	Insulins and analogues	28 (23.93)
A10B	Blood glucose lowering drugs, excl insulins	26 (22.22)
A10BA02	Metformin	1 (0.85)
A10BX02	Repaglinide	6 (5.13)
A10BH01	Sitagliptin	2 (1.71)
A10BH02	Vildagliptin	3 (2.56)
A10BH05	Linagliptin	12 (10.26)
B01	Antithrombotic agents	110 (94.02)
C01	Cardiac therapy	18 (15.38)
C03	Diuretic	72 (61.54)
C02	Antihypertensives	97 (82.91)
C02CA04	Doxazosin	48 (41.03)
C08CA11	Manidipine	49 (41.88)
C07AB03	Atenolol	27 (23.08)
C07AB07	Bisoprolol	24 (20.51)
C09CA01	Losartan	11 (9.40)
C10	Lipid modifying agents	70 (59.83)
C10AA05	Atorvastatin	39 (33.33)
C10AA01	Simvastatin	25 (21.37)
C10AX09	Ezetimibe	16 (13.68)
C10AX06	Omega-3-triglycerides incl. other esters and acids	18 (15.38)
D	Dermatologicals	5 (4.27)
M04A	Antigout preparations	55 (47.01)
N02	Analgesics	19 (16.24)
N03	Antiepileptics	11 (9.40)
N05	Psycholeptics	13 (11.11)
N06	Psychoanaleptics	14 (11.97)
R	Respiratory system	28 (23.93)
L04	Immunosuppressants	37 (31.62)
J07BN	COVID-19 vaccines	115 (98.29)

^a^ The list of medications is exhaustive for our cohort and was identified as documented in the medical records. ^b^ Unless otherwise indicated: numbers present *n* (%).

**Table 8:** Factors influencing quantitative ineffectiveness in KRT patients.

Factors	Category	Quantitative Ineffectiveness (Prescribed Quantity or Dosage of a Medication is Insufficient)		OR	95% CI	*p*-Value
		No	Yes			
Risk of adverse effects	No	2 (16.7%)	10 (83.3%)	1		
	Yes	3 (2.9%)	102 (97.1%)	1.802	0.076–18.631	0.6481
Undertreated conditions	No	2 (66.7%)	1 (33.3%)	1		
	Yes	3 (2.6%)	111 (97.4%)	23.883	1.265–1062.966	0.0445
Anemia	No	3 (25.0%)	9 (75.0%)	1		
	Yes	2 (1.9%)	103 (98.1%)	8.836	0.851–90.667	0.0524

## Data Availability

For data supporting the reported results, contact the corresponding author.
